# Cerebral Vascular Toxicity of Antiretroviral Therapy

**DOI:** 10.1007/s11481-019-09858-x

**Published:** 2019-06-17

**Authors:** Luc Bertrand, Martina Velichkovska, Michal Toborek

**Affiliations:** Department of Biochemistry and Molecular Biology, University of Miami Miller School of Medicine, Gautier Bldg., Room 528, 1011 NW 15th Street, Miami, FL 33136 USA

**Keywords:** Antiretroviral therapy, Blood-brain barrier, Cerebrovascular toxicity, Mitochondria, Neurotoxicity

## Abstract

HIV infection is associated with comorbidities that are likely to be driven not only by HIV itself, but also by the toxicity of long-term use of antiretroviral therapy (ART). Indeed, increasing evidence demonstrates that the antiretroviral drugs used for HIV treatment have toxic effects resulting in various cellular and tissue pathologies. The blood-brain barrier (BBB) is a modulated anatomophysiological interface which separates and controls substance exchange between the blood and the brain parenchyma; therefore, it is particularly exposed to ART-induced toxicity. Balancing the health risks and gains of ART has to be considered in order to maximize the positive effects of therapy. The current review discusses the cerebrovascular toxicity of ART, with the focus on mitochondrial dysfunction.

**Graphical Abstract:**
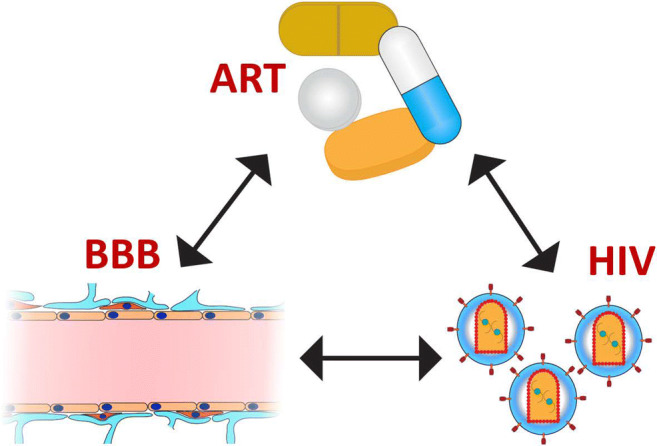
Graphical representation of the interactions between HIV, antiretroviral therapy (ART), and the blood-brain barrier (BBB).

Graphical representation of the interactions between HIV, antiretroviral therapy (ART), and the blood-brain barrier (BBB).

## Introduction

The introduction of combined anti-retroviral therapy (ART) has changed the outcome and prognosis of HIV infection. What was once a fatal disease is now controlled, and the infected patients survive longer. In these long-term survivors, several pathologies are observed, such as cardiovascular, lipid, metabolic, and neurological disorders (Clifford and Ances 2013; Deeks et al. 2013; Galescu et al. 2013; Kebodeaux et al. 2013; Lake and Currier 2013; Bertrand et al. 2019b). Before the advent of ART, neurological disorders in HIV patients were often associated with severe cognitive dysfunction, such as HIV-associated-dementia. Currently, neurological disorders are rather associated with mild and slow progressive degeneration of cognitive and motor functions (Clifford and Ances 2013); this susceptibility is correlated with age (Becker et al. 2004). While persistent (albeit at low rates) HIV replication in the brain may be responsible for neurocognitive alterations observed in infected individuals, the toxicity of anti-retroviral drugs is also likely to contribute to neurodegenerative disorders in HIV patients.

Antiretroviral drugs have restricted capability of crossing the blood-brain barrier (BBB) and reaching therapeutic concentrations in the CNS. Several efflux pumps, such as P-glycoprotein (P-gp) and organic anion transporters, can actively remove these therapeutics out of the CNS. In addition, antiretroviral drugs that are highly bound to plasma proteins are less likely to penetrate the BBB. Low molecular weight and hydrophobicity of drugs are factors that promote BBB penetration, while ionization has a negative effect. In addition, multiple mechanisms can play a role in the ability of drugs to cross into the brain parenchyma. These include the paracellular aqueous pathway, the transcellular lipophilic pathway, transport proteins, receptor mediated transcytosis and adsorptive transcytosis (Bertrand et al. 2016a). Because of these factors, antiretroviral drugs are frequently not reaching effective therapeutic concentrations in the brain, contributing to the development of drug resistance (Ferretti et al. 2015) and/or formation of HIV reservoirs in the CNS. Nevertheless, their concertation in the plasma is sufficient to induce toxicity to the brain vasculature. The present review describes the toxicity of antiretroviral drugs and their impact on the development of neurological disorders in the context of the BBB and cerebral vascular biology.

## Status of BBB in HIV Infected Patients

Following infection by HIV, the virus quickly propagates and infects several tissues, including the brain. It has been demonstrated that HIV reaches the CNS shortly after infection, infects multiple cell types and can also act as a reservoir (Sturdevant et al. 2015; Oliveira et al. 2017; Hsu et al. 2018). However, a route by which HIV crosses the BBB and disseminates in the CNS remains debated (Toborek et al. 2005). After successful initiation of anti-retroviral therapy (ART), the virus typically becomes undetectable in the plasma of patients, and the main signs of infection subside, including CD4+ T cell depletion. Nevertheless, a low level of HIV activity persists despite successful therapy, and some toxic viral proteins, such as gp120 and Tat, are still present, albeit at very low concentrations (Martinez-Picado and Deeks 2016; Ahmed et al. 2018). The fact that HIV patients, even those who adhere to therapy, are still at a higher risk of developing co-morbidities, such as metabolic and cardiovascular diseases (Chow et al. 2012; Warriner et al. 2014; Farhadian et al. 2017) raises a question about the underlying factors contributing to the development of these disorders.

**Fig. 1:**
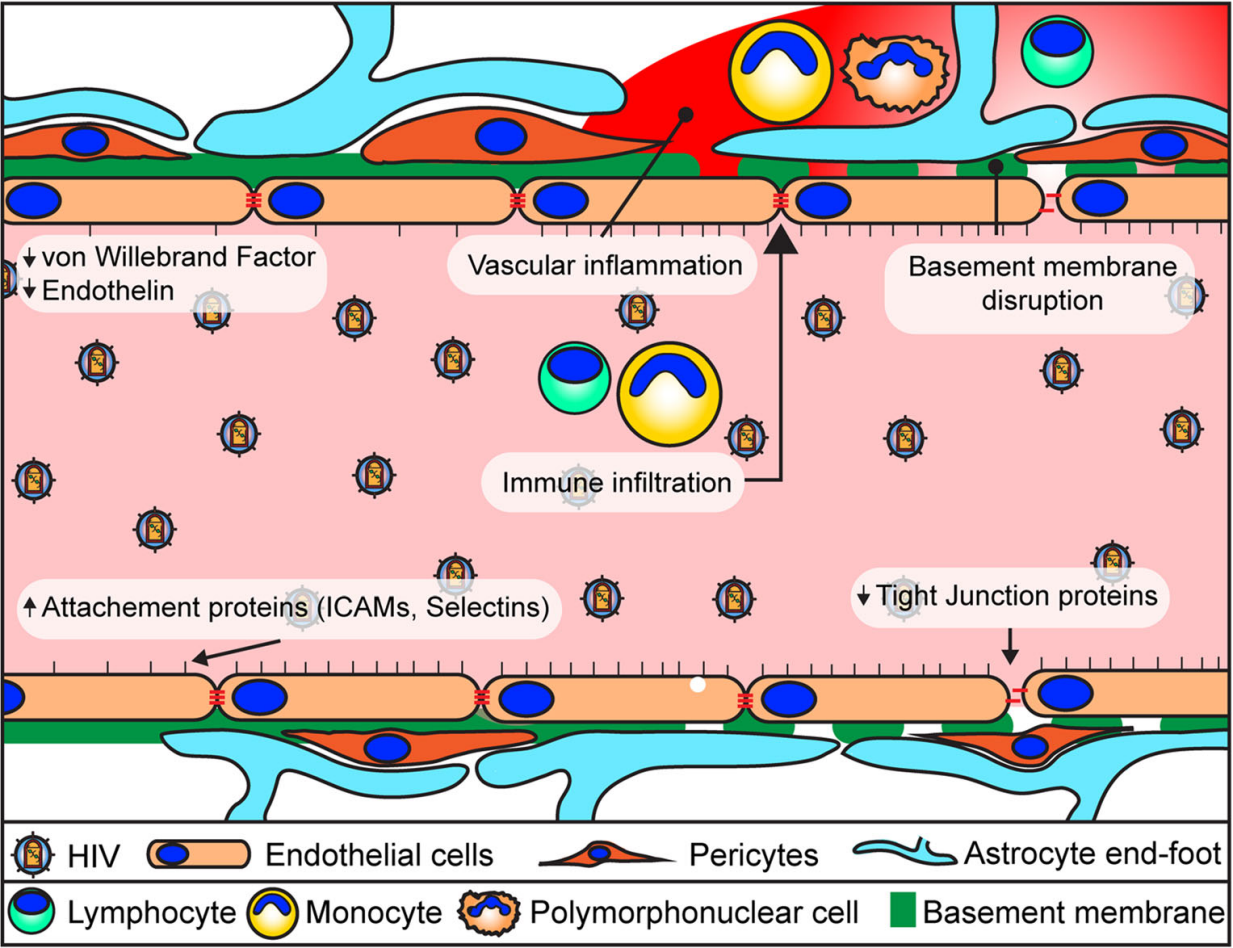
**Blood-brain barrier dysfunction in HIV-infected patients.** Visual representation of the mechanisms leading to BBB disruption in HIV infection

Alterations of the BBB occur in the earliest stages of infection (Li et al. 2013; Peluso et al. 2013; Wright et al. 2015) and then persist throughout the infection (Fig. 1). This dysfunction is likely linked to the several co-morbidities observed in persons living with HIV, such as cerebrovascular disease and neurocognitive problems. The viral envelope gp120 can cause endothelial cell senescence and induce the expression of stress fibers (McRae 2016; Hijmans et al. 2018). HIV Tat is another viral protein that has potent toxicity. It can affect BBB integrity and tight junction protein assembly in brain microvascular endothelial cells via a process that has been linked to signaling via small GTPases and ERK1/2 (Pu et al. 2005; Zhong et al. 2012). In addition, Tat exposure can lead to elevated intracellular reactive oxygen species (ROS) levels and cause apoptosis (Toborek et al. 2003). Indeed, vascular endothelial cells are very susceptible to oxidative damage (Toborek et al. 1995; Lee et al. 2001, 2004). Finally, viral proteins Nef and Vpr have also been shown to be associated with BBB permeability and neurotoxicity (Ferrucci et al. 2013; Saribas et al. 2015). There are also signs of a persistent inflammation in the vasculature of people living with HIV. In addition to aforementioned viral factors, elevated levels of ROS and increased circulating levels of proinflammatory molecules, such as TNF-α, IL-1β, and C-reactive protein, can contribute to underlying chronic inflammation in infected individuals (Brabers and Nottet 2006; Ross et al. 2009; Younas et al. 2016). These changes are accompanied by an increase in adhesion molecules on the brain endothelium, such as VCAM-1, ICAM-1, P-selecting, and platelet endothelial cell adhesion molecule (PECAM-1 or CD31) (Dhawan et al. 1997; Wolf et al. 2002; Bertrand et al. 2019b). The accumulation of these inflammatory mediators can further lead to the development on vasculopathies associated with HIV infection (Chetty 2001). These changes result in a leaky barrier, impacting proper functioning of endothelial cells and resulting in dysfunction of astrocytes and pericytes (Ahmed et al. 2018). There is a clear indication that following HIV-1 infection, the integrity of the BBB is gradually affected.

## Impact of BBB Function on Efficiency of HIV Treatment

While the focus for treatment of HIV is aimed at peripheral replication in immune cells, mounting evidence demonstrates that the CNS is an important HIV-1 reservoir that will need to be addressed for increasing patient wellness and curative approaches. The BBB plays a pivotal role in the maintenance of the CNS. It controls the inflow of nutrients, while at the same time actively pumps out metabolic byproducts and toxic compounds (Cornford and Hyman 2005; Hawkins et al. 2006; Qosa et al. 2015). This strict control protects the brain; however, it also provides a severe hindrance to overcome when treatment molecules need to reach the CNS. Many of the drugs employed to treat HIV do not readily cross the BBB, some are even actively pumped out by transporters such as P-gp and/or multidrug resistance protein 1 (MRP1) (Robillard et al. 2014). This results in suboptimal drug concentrations in the CNS that cannot completely suppress viral activity and may contribute to the selection of resistant mutations. This partial viral suppression allows for the synthesis of viral proteins that contribute to viral propagation, potentiate ART toxicity, and lead to CNS comorbidities (Shah et al. 2016; Stern et al. 2018).

Several strategies have been employed to overcome BBB restrictions for efficient neuroHIV treatment (Bertrand et al. 2016a). Increasing treatment dose for some therapeutics can help attain sufficient CSF concentration to block viral activity. However, potential toxicity can cause a problem for some anti-retroviral drugs. Protease inhibitors are highly targeted by efflux pumps, such as P-gp; therefore, the current strategy is to saturate their activity with ritonavir, which has higher affinity to this transporter (Drewe et al. 1999; Marzolini et al. 2013). Several new approaches are being developed to increase the translocation capacity by modification of existing drugs, such as coupling molecules to transferrin in order to use its receptor to facilitate transport (Clark and Davis 2015; Gu et al. 2017), or attaching drugs to cell penetrating peptides (Kamei et al. 2018; Yuan et al. 2019). Finally, nanoparticles are also being used to carry therapeutic agents across the BBB (Belgamwar et al. 2018; Roy et al. 2018). While several of these strategies have been successful in overcoming BBB limitations, there is also a concern that these new elements could themselves be toxic (Ajdary et al. 2018).

## ART-Induced BBB Dysfunction

**Fig. 2:**
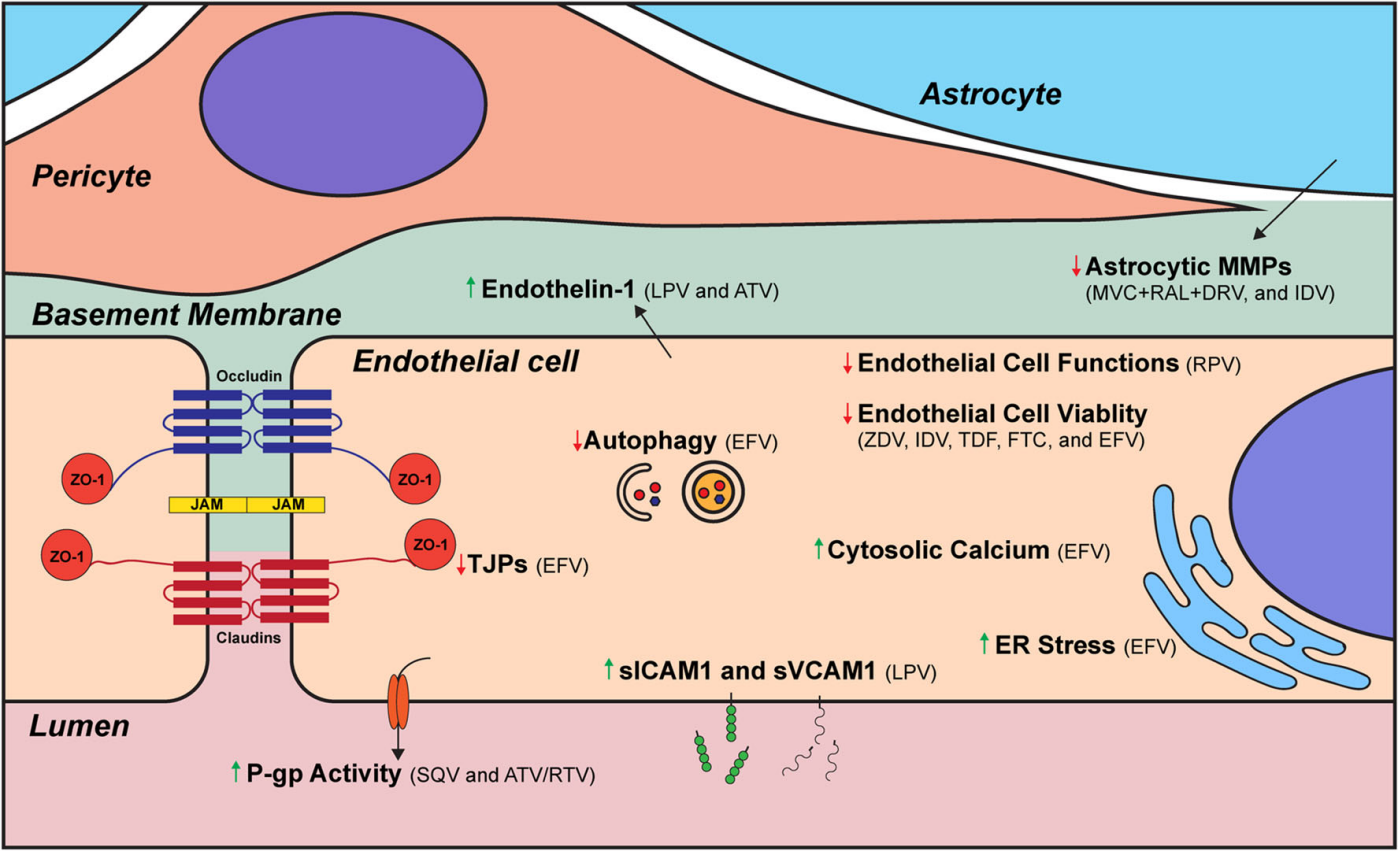
**Alterations of BBB functionality as the result of exposure to antiretroviral drugs.** Alterations (red arrow: decrease; green arrow: increase) of cellular processes at the level of the BBB resulting from exposure to antiretroviral drugs. (LPV: lopinavir; EFV: efavirenz; SQV: saquinavir; ATV: ataznavir; RTV: ritonavir; ZDV: zidovudine; IDV: indinavir; TDF: tenofovir; FTC: emtricitabine; MVC: maraviroc; RAL: raltegravir; DRV: darunavir)

As previously stated, the presence of HIV can compromise the integrity of the BBB and affect the normal function of the cells which compose this barrier (Toborek et al. 2005). In addition, the majority of people living with HIV undergo ART treatment. This results in an almost complete absence of viral activity, at least at the periphery. However, due to the nature of HIV infection, patients need to continue to take medications for the rest of their lives. Given this fact, ART toxicity needs to be analyzed thoroughly, since side effects can accumulate over time and cause serious repercussions to patients, including dysfunction of the BBB (Fig. 2). Indeed, alterations of the BBB integrity have been reported in several longitudinal studies that observed an initial improvement of endothelial cell function following the initiation of ART, but dysfunction eventually returned (Wolf et al. 2002; Shankar and Dube 2004; Haser and Sumpio 2017). This was further revealed when comparing patients with similar treatment outcomes (HIV copy numbers and NADIR) using two different ART regimens. A study observed that the levels of endothelial progenitor cells and vascular inflammation in patients treated for 24 weeks using Darunavir were indistinguishable from non-infected patients, while those exposed to Rilpivirine showed signs to endothelial cell dysfunction (Echeverria et al. 2017). Alterations of the health of cerebral vasculature, evidenced by the detection of cerebral small vessel disease (CSVD), has been observed in patients exposed to ART, especially those treated with protease inhibitors (Morgello et al. 2014; Soontornniyomkij et al. 2014). More recently, a study has found evidence that Emtricitabine can be linked to the development of CSVD in forebrain white matter (Soontornniyomkij et al. 2018). Experimental studies also demonstrated that Efavirenz can have deleterious effects on ischemic stroke, leading to increased tissue damage and BBB deterioration (Bertrand et al. 2016b). On the other hand, administration of low toxicity ART is beneficial for the outcome of stroke when compared to the untreated HIV-infected group (Bertrand et al. 2019b). An additional factor to take into consideration is that co-morbidities can be exacerbated by ART toxicity. As such, a recent investigation demonstrated that hyperglycemia exacerbates endothelial cell viability upon exposure to a combination of Zidovudine and Indinavir (Prasad et al. 2016).

A feature of the current HIV epidemiology is an increase in aging of infected patients (Cysique and Brew 2014; Pathai et al. 2014). A recent study indicated based on the epigenetic clock, that HIV infection led to an average aging advancement of 4.9 years, increasing expected mortality risk by 19% (Gross et al. 2016). This problem is of great significance because ~70% of adults with HIV in the US are likely to be 50 or older by the year 2020 (Aging 2013). The life expectancy of a 20-year-old HIV-positive adult on ART is expected to be ~70 years (Antiretroviral Therapy Cohort 2008; Samji et al. 2013). These factors can have a huge impact on ART as drug toxicity can change with age. Firstly, altered pharmacokinetics can be observed in older patients due to several shifts in body functions (Winston and Underwood 2015). Impaired gastric functions can affect adsorption rate, leading to an increase in systemic levels of drugs such as Rilpivirine (Marzolini et al. 2011). Body composition, such as percentages of fat, lean muscle, and albumin concentration can result in altered drug distribution, namely higher concentration in certain tissues (Cooperman et al. 2007). Finally, a reduction in liver and renal functions can affect drug metabolism and clearance, leading to a bioaccumulation of all class of anti-retroviral drugs (Crawford et al. 2010; Baxi et al. 2014).

As indicated above, the integrity of the BBB is reliant on the relationship of multiple partners, especially endothelial cells, pericytes and astrocytes (Bertrand et al. 2019a). Given the importance of cell-cell communication in the neurovascular unit (Cho et al. 2017), toxicity reported on one cell type is expected to affect BBB functions, which is why toxicity of ART on different cell types needs to be considered.

HIV patients on ART exhibit changes in endothelial functions, such as lower expression levels of von Willebrand Factor or protein S (Chwiki et al. 2017). A combination of Tenofovir and Emtricitabine can act as cellular stressors, leading to endothelial cell senescence, as demonstrated by a reduction in proliferation, and an increase in inflammatory markers (Cohen et al. 2018). This results in decreased BBB integrity and impaired endothelial cell functions. Exposure to Efavirenz has been shown to reduce endothelial viability at relatively low concentrations. This effect has been linked to multiple insults, such as a dysregulation of polymerase γ function, imbalance of intracellular calcium levels and depletion of ADP (Bertrand and Toborek 2015; Weiss et al. 2016; Faltz et al. 2017). The latter can result in overactivation of the DNA repair enzyme poly ADP polymerase (PARP), which leads to a loss of cell viability and necrotic cell death. It was recently reported that Lopinavir exposure can lead to an increase in sICAM-1, endothelin-1, and sVCAM-1, which are proinflammatory and can impair cerebral blood flow (Mata-Marin et al. 2013; Auclair et al. 2014). Exposure of endothelial cells to Efavirenz can severely impact BBB integrity by decreasing levels of tight junction proteins claudins-1/5, occludin, ZO-1, and JAM-1. This results in an increase in permeability, a phenomenon which has been shown both in vivo and in vitro (Bertrand and Toborek 2015; Bertrand et al. 2016b; Faltz et al. 2017). Furthermore, it can affect cellular stress response by disrupting ER stress and autophagic responses.

Matrix metalloproteinase (MMP) activities are tightly regulated in the BBB and increased levels can cause leakiness, while reduced levels can affect angiogenesis and plasticity (Huang et al. 2011). A recent study demonstrated that exposure of astrocytes to Maraviroc, Raltegravir and Darunavir at high concentrations can lead to a reduction in MMP synthesis and activation. This effect was also shown to synergize when drugs were combined (Latronico et al. 2018). Indinavir was demonstrated to inhibit the conversion of ProMMP2 into active MMP2 and decrease angiogenesis (Barillari et al. 2014).

Efflux and influx transporters are important to BBB function as they regulate the flow of molecules in and out of the CNS. Dysregulation of their activity can lead to several complications, such as a reduction in nutrient import, and increase in the removal of anti-retroviral drugs. Activity of the transporter P-gp has been shown to increase in response to exposure to Saquinavir and HIV-1 (Roy et al. 2013). This increase was also observed in response to a combination of Ritonavir and Ataznavir. The latter effect has been linked to an increase in expression of the pregnane X receptor and the constitutive androstane receptor (Chan et al. 2013).

The impact of ART-mediated cerebral vascular toxicity can be compounded or further amplified by chronic inflammation present in the BBB due to HIV infection. As discussed, chronic inflammation occurs at the BBB in HIV patients (Wolf et al. 2002; Kamtchum-Tatuene et al. 2019). Several antiretroviral drugs, such as Nelfinavir, Efavirenz, and Zidovudine, have been implicated in stimulation of inflammatory responses, including leukocyte adhesion, in a process linked to elevated oxidative stress (Mondal et al. 2004). Combined with increased release of chemokines, this process can further stimulate infiltration of brain parenchyma with monocytic cells (Park et al. 2001), contributing to inflammation and potentially increasing HIV-1 entry into the CNS.

As indicated above, there is a large body of evidence demonstrating that several of the drugs used in HIV-1 treatment can have detrimental effects on the health of the BBB. While the benefits of ART are undeniable, and the improvement to patients’ health with the control of HIV far outweighs the side effects of these therapeutics, efforts need to be continued to improve these therapies and decrease drug toxicity. This can be accomplished through the in-depth analysis of potential side effects of ART and the continued search for new therapy avenues.

## ART Toxicity: Beyond the BBB and Polymerase Gamma

It is now recognized that the BBB plays a critical role in maintaining the pool of neuronal progenitor cells in the brain and in proper neurogenesis (Williams et al. 2014). Indeed, the neurogenic niches are localized around the brain microvasculature. Approximately 47% of dividing neural progenitor cells (NPC) and 46% of transit amplifying cells (i.e., cells that give rise to neuroblasts) are located within 5 μm of the endothelium (Shen et al. 2004, 2008). These progenitors can directly contact the endothelium in the areas lacking astrocyte end-feet and pericyte coverage, suggesting that the brain endothelium is an essential matrix and source of external cues for NPCs (Shen et al. 2004, 2008; Teng et al. 2008). The brain endothelium is believed to create a microenvironment that mediates progenitor cell trafficking and differentiation by providing external signage as guidance cues. One of these mechanisms is the binding of endothelium-produced CXCL12 to CXCR4 on NPCs for enhanced attachment of NPCs to endothelial cells, which then provide growth factors for NPC survival and proliferation (Williams et al. 2014). Thus, the integrity of the BBB is vital for normal self-renewal, proliferation, and survival of NPCs (Palmer et al. 2000; Shen et al. 2004; Ramirez-Castillejo et al. 2006; Riquelme et al. 2008; Park et al. 2013).

Because of the close interactions between the BBB and NPCs, we extended our studies on ART-induced dysfunction of the brain endothelium to other cells of the neurovascular unit and/or the cells that are located in the immediate proximity of the BBB. Our recently published study indicated that exposure to ART causes increased reactive oxygen species (ROS) generation which results in mitochondrial dysfunction, thus promoting cellular senescence (Velichkovska et al. 2018). The study employed several ART combinations, such as Tenofovir and Emtricitabine (both nucleoside reverse transcriptase inhibitors, NRTIs), Tenofovir, Emtricitabine, and Raltegravir (NRTIs plus a protease inhibitor), and Tenofovir, Emtricitabine, Ritonavir, and Darunavir (NRTIs plus integrase inhibitors). The majority of these combinations induced mitochondrial dysfunction. Exposure to Tenofovir, Emtricitabine, Ritonavir, and Darunavir resulted in a 37% increase in beta-galactosidase staining and shortening of telomere length to more than half of the length of controls, indicating accelerated NPC senescence in response to ART exposure.

**Fig. 3:**
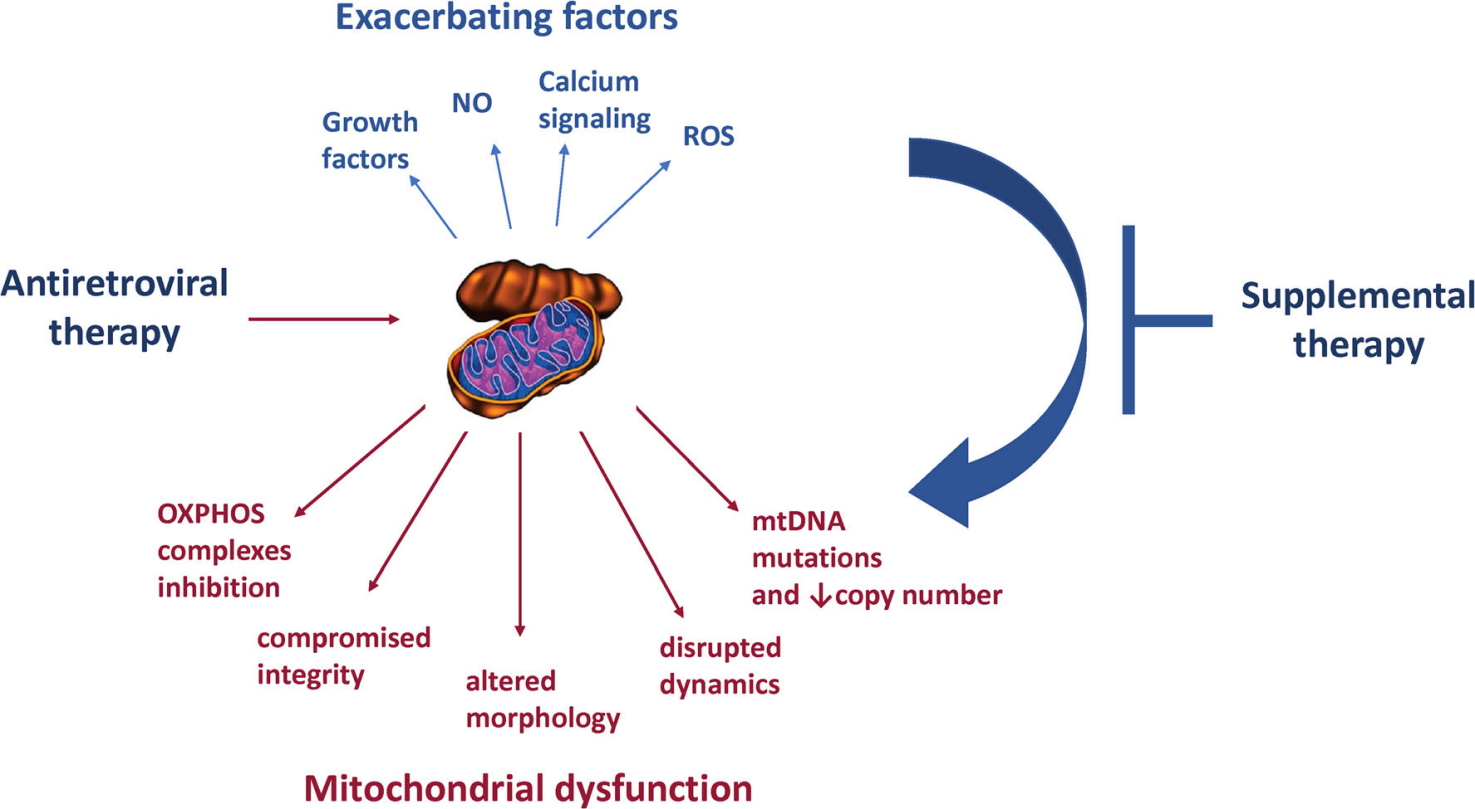
**Factors contributing and exacerbating mitochondrial dysfunction as the result of exposure to antiretroviral drugs.** Supplemental therapy targeting the exacerbating factors can prevent the onset of mitochondrial dysfunction

A large body of evidence indicates that the toxic side effects of ART include a mitochondrial dysfunction component (Fig. 3). However, the specifics of the underlying mechanisms are yet to be elucidated. It is well established that polymerase gamma is affected by reverse-transcriptase inhibitors designed to inhibit the action of reverse transcriptase (Lewis and Dalakas 1995). Since polymerase gamma is the mitochondrial polymerase replicating mitochondrial DNA (mtDNA), its inhibition can introduce mtDNA mutations which then induce mitochondrial dysfunction or results in a decrease in overall mtDNA levels. However, ART can induce additional alternations of mitochondrial morphology and function, suggesting induction of multiple independent toxic mechanisms; especially under the administration of different classes of antiretroviral drugs or drug mixtures. Moreover, feedback response programs could be connecting one type of mitochondrial dysfunction to another; but their existence, activation, and regulation are poorly understood and require further investigation.

Toxic effects of antiretroviral drugs on mitochondria via mechanisms beyond inhibition of polymerase-gamma have been reported as early as the 1990s. For example, dose-dependent inhibition of NADH-linked respiration has been reported under AZT administration across multiple tested tissues. An inhibition of electron transport chain function was suggested to be responsible for these effects because complex I inhibition is responsible for NADH oxidation (Modicanapolitano 1993). Additionally, inhibition of succinate-linked respiration and uncoupled electron transport chain have been specifically reported for brain tissue. However, the mechanism through which AZT inhibits complex I or succinate-linked respiration is still not clear. Antiretroviral drug induced polymerase-gamma inhibition follows the same mechanisms as those designed against reverse-transcriptase. However, the aforementioned additional mitochondrial targets reveal that there are off target mechanisms which can be induced by antiretroviral drugs, making the acquisition of better knowledge in this field more challenging.

In addition to AZT, other antiretroviral drug types have also been shown to impact electron transport chain function through Complex I inhibition. Namely, the non-nucleoside reverse-transcriptase inhibitor, Efavirenz, was found to inhibit Complex I in primary rat neuron cultures (Blas-García et al. 2010), as well as human neurons and glia (Apostolova et al. 2015a). Furthermore, mitochondrial complex I gene expression was found to decrease in neurons treated with Didanosine (Zhu et al. 2007). Complex I is the first component of the mitochondrial electron transport chain which has a crucial role in proper mitochondrial function and ATP production output. Inhibition of its function has in fact been associated with the pathogenesis of neurodegenerative diseases (Schapira 2010). A commonly used research method to estimate electron transport chain function is measuring the oxygen consumption rate (OCR), and we (and others) have shown that ART drug combinations induce alternations of OCR (Apostolova et al. 2015a; Cohen et al. 2017; Velichkovska et al. 2018). An interesting observation also indicates that the inhibition of Complex I and alterations to the electron transport chain function may occur without any changes in the OCR levels (Ciavatta et al. 2017). The explanation for this phenomenon was suggested to arise from the activation of Complex II to act as a bypass and substituting for the Complex I electron-pumping function. In fact, upregulation of the remaining complexes function was reported as well. Cerebral rat mitochondria treated with AZT showed decreased Complex I activity, while in parallel had dose-dependent increases of Complex II and Complex IV (Ewings et al. 2000). Hence, these compensatory mechanisms could result in maintained unaltered net OCR levels. All of this data taken together raises the concern that even though the OCR might be temporarily maintained, the altered functions of individual complexes can have other negative implications, especially over lifetime exposures to ART. Additionally, a comparison of these findings to the aforementioned studies that reported OCR alternations suggests that there are multiple mechanisms in which electron transport chain function can be affected by ART. Indeed, no changes in viability were found in cells exposed to selected anti-retroviral drugs through the reductive-dependent reagent, CTB (Ciavatta et al. 2017). These results suggest the existence of a non-mitochondrial mechanism compensating for the lost mitochondrial reductive capacity. Nonetheless, similar to the electron transport chain function, it is questionable if such compensation mechanisms can be retained over long-time exposures to ART, which is required to control the HIV infection.

With respect to mitochondrial integrity, changes in the neurotransmitter levels N-acetylaspartate (NAA) upon ART exposure have been reported in several studies (Schweinsburg et al. 2005; Winston et al. 2010; Young et al. 2014; Sailasuta et al. 2016). NAA is associated with loss of mitochondrial integrity because it correlates to ATP inhibition and decreased OCR levels (Bates et al. 1996). Additionally, its signal was shown to decrease under inhibition of the succinate dehydrogenase step of the tricarboxylic cycle (Demougeot et al. 2001). NAA is also a neuron-specific indicator (Moffett et al. 1991; Simmons et al. 1991) and was reported to be an axon-specific marker (Bjartmar et al. 2002). HIV patients taking antiretrovirals were found to have the highest decrease in NAA in their frontal white matter compared to the group of non-infected individuals, while the HIV patients who did not receive treatment had a milder decrease (Schweinsburg et al. 2005). Additionally, the higher number of antiretrovirals administered, the higher the decrease; which is particularly alarming since all antiretrovirals are used as part of drug mixtures, whereas a lot of studies on antiretroviral drugs toxicity in general report findings for individual drugs. The decreasing levels of NAA may also imply that there is a decrease in the total number of mitochondria since NAA is produced by energy-dependent reactions inside mitochondria, and there have been no mechanisms identified for upregulation of NAA synthesis when its concentration decreases (Schweinsburg et al. 2005). However, it is also possible that NAA levels decrease due to a dysfunctional electron transport chain and a decrease in ATP. Even if this is the only mitochondrial parameter affected at an earlier point, it could make mitochondria more prone to developing compromised functions as the result of prolonged ART exposure. Recently, an untargeted CSF metabolite analysis based on a machine learning model identified NAA as one of the key CSF metabolites that can be used to predict incidence of HAND. Other identified key-classifiers were glutamate, ketone bodies, and markers of glial activation, all of which emphasize the importance of mitochondrial function, oxidative stress, and metabolic waste pathways in association with HAND onset (Cassol et al. 2014). Furthermore, a structural MRI study comparing the different levels of progression of HAND found that NAA was decreased in patients that had HIV-associated dementia and in patients that were still asymptomatic in comparison to the group that was neurocognitively unimpaired (Alakkas et al. 2018).

Mitochondrial morphology appears to be profoundly affected by HIV and antiretrovirals as well. Part of the evidence for this mechanism was generated in studies on vertical transmission of HIV and antiretroviral toxicity. Infants that were exposed to NRTI through their mothers before birth had minimal effects on the function of the oxidative phosphorylation complexes; however, their mitochondrial morphology was significantly altered. Hence, altered morphology appears to be among the most vulnerable mitochondrial parameters affected by ART (Divi et al. 2010). A more recent study on perinatal exposure to ART (Zidovudine [AZT]/Lamivudine [3TC]/Abacavir, or AZT/3TC/Nevirapine drug combinations) has confirmed mitochondrial morphology alternations in the heart and brain that persist in *Erythrocebus patas* monkeys until the age of 3 (Liu et al. 2016). Furthermore, only the former ART combination induced oxygen consumption rate changes, while only the latter induced mtDNA changes; emphasizing the possibility of distinct toxicity mechanisms.

There is evidence that infants under vertical exposure to antiretrovirals experience developmental changes later in life (Benki-Nugent et al. 2015) due to alterations in tissues that are sensitive to mitochondrial dysfunction (Sibiude et al. 2015a). Examples of such metabolic changes include cardiac dysfunction (Sibiude et al. 2015b) or neurodevelopmental delays, even with successful viral repression (Strehlau et al. 2016). These observations raise the possibility that the alternations of mitochondrial morphology discussed above might be an early sign of mitochondrial stress that develops into pathology as a function of time. Additionally, the impact on mitochondrial morphology could lead to depletion of the mitochondrial count per cell which may have a negative impact on meeting energy demands (Schweinsburg et al. 2005).

It is not clear how mitochondrial morphology changes are linked to functional alternations. Morphological alterations were found to be affected by all tested NRTIs, NNRTIs, and PIs (Robertson et al. 2012); but interestingly, the extent of these alterations did not correlate to the levels of neurotoxicity of the drugs or the level to which they altered the mitochondrial membrane potential. Additionally, mitochondrial morphological changes were observed in cases when there was no detection of mtDNA mutations and/or changes in oxidative phosphorylation proteins (Liu et al. 2016), and when the level of polymerase gamma inhibition of antiretroviral drugs was compared to the efficacy of the electron transport chain, no correlation was found (Hung et al. 2017). The impact of ART on mtDNA or the electron transport chain was also reported in the absence of any mitochondrial morphology alternations (Ewings et al. 2000; Gerschenson et al. 2004). Nevertheless, it might be too early to conclude that all of these mechanisms are completely independent. It is well known that altered mitochondrial morphology could imply changes in the rates of fission and fusion of mitochondria, or the regulation of apoptosis (Karbowski and Youle 2003). Additionally, it was reported that a cytochrome c oxidase mutation can induce changes in mitochondrial morphology in order to activate a mechanism of communication between mitochondria inside the cell, enabling them to exchange their genetic material and proteins in order to retain overall function (Nakada et al. 2001). In fact, we and others have also shown mitochondrial transfer to be a stress-response mechanism activated by HIV infection in cells of the BBB (Castro et al. 2017). However, there is no evidence to suggest the existence of such a protective mechanism in the context of antiretroviral-drug induced mitochondrial dysfunction, hence, it is not clear if the changes in mitochondrial morphology are induced by an injury to the mtDNA or are a result of an independent mechanism.

Exposure to ART can also induce alternations of mitochondrial dynamics. For example, exposure to Efavirenz as well as Tenofovir and Dideoxyinosine resulted in a significant decrease in MAP2 staining (Ciavatta et al. 2017). The microtubule-associated protein 2 (MAP2) is important for neurite maintenance, growth, and extension; hence, it is primarily used to track morphological and developmental alternations of dendrites, which were also reported to occur as a result of several different ART drugs exposures (Robertson et al. 2012; Akay et al. 2014). Importantly, MAP2 binds to the outer membrane of mitochondria (Lindén et al. 1989) and altered MAP2 also implies changes in mitochondrial dynamics. MAP2 as a scaffolding protein is crucial for the transport of mitochondria along axons, and this process has been recognized to be dysfunctional in many neurodegenerative diseases (Su et al. 2010). Altered mitochondrial dynamics may either induce or exacerbate mitochondrial dysfunction during neurodegeneration since this process has an impact on mitochondrial bioenergetics and integrity of the mitochondrial genome, in addition to controlling cell death and synaptic maintenance, supporting a notion that neurotoxic effects of Efavirenz may be mediated in part through synaptic damage (Tovar-y-Romo et al. 2012).

In addition to the impairment of the electron transport chain and mitochondrial morphology, exposure to anti-retroviral drugs can affect glucose metabolism. Evidence also indicates that not the parent drugs, but their metabolites may be ultimately responsible for mitochondrial toxicity, emphasizing the importance of drug metabolism. Indeed, stimulation of glycolytic flux in cultured astrocytes was observed due to exposure to the primary metabolite of Efavirenz (Brandmann et al. 2013), and an earlier study reported that HIV patients treated with Efavirenz displayed lactic acidosis (Chow et al. 2007). Interestingly, the reported increase in glycolytic flux was independent of oxidative phosphorylation activity, since no additive effect was found under the inhibition of Complex I or Complex II (Brandmann et al. 2013). Moreover, in vitro experiments in astrocytes found this effect to be specifically induced by Efavirenz’s primary metabolite but not the parent compound. There is also evidence for the connection between drug metabolism and extent of mitochondrial toxicity for other antiretrovirals. A recent study on HIV positive patients taking the thymidine analogue Stavudine, found a connection between the occurrence of mitochondrial toxicities such as sensory neuropathy and the genetic variation in thymidine synthesis pathways and the analogue transport and metabolism (Moketla et al. 2018).

## Different Cells, Different Vulnerabilities

An important aspect of the mechanisms underlying mitochondrial toxicity is the fact that they do not completely overlap within different brain regions; rendering some cell types more vulnerable than others. For instance, several studies have reported the cells in the cerebrum to be more vulnerable to mitochondrial ART toxicity compared to cells in the cerebellum (Ewings et al. 2000; Zhang et al. 2014). Ewing et al. found that mitochondria from the cerebrum were impaired and their functions of oxidative phosphorylation complexes were altered by AZT. However, none of these alternations were observed in mitochondria from the cerebellum. Additionally, HIV patients on Stravudine or Didanosine exhibited alterations of NAA levels only in the frontal white matter, but not in the frontal grey matter (Schweinsburg et al. 2005). mtDNA levels were also unaltered when analyzing the whole brain tissue, and changes were detected only when cortical neurons were isolated (Zhang et al. 2014). These alterations were specifically identified as mutations of the mtDNA D-loop (Zhang et al. 2015). Additionally, the Didanosine-induced decrease in mtCOXI expression observed in neurons was not found to be present in Schwann cells (Zhu et al. 2007). A study that compared Efavirenz’s effects on neuron and astrocyte primary cultures (Funes et al. 2014) indicated that mitochondrial membrane potential and ATP levels were significantly affected in both astrocytes and neurons upon ART exposure; however, only astrocytes could activate the adenosine monophosphate-activated protein kinase and therefore compensate for the diminished ATP levels by upregulating glycolysis. Such events can result in glial cells survival and neuronal degeneration. The protective glycolysis activation in glial cells was also reported by other groups (Brandmann et al. 2013). Interestingly, selected neuronal populations appear to be more vulnerable than others to toxicity of ART. A recent study comparing the effects of ten different antiretrovirals with high BBB penetration scores found that cortical nerve termini were not affected, while striatal nerve terminals exhibited reduced mitochondrial spare respiratory capacity for all drugs testes. The experiments also indicated that this connection was dose-dependent and resulted in depletion of ATP at synapses (Stauch et al. 2017).

**Fig. 4:**
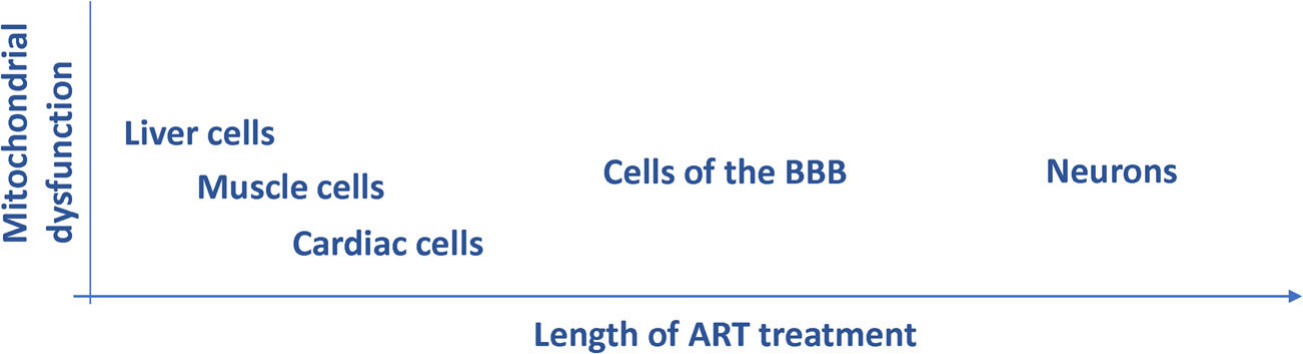
Onset of mitochondrial dysfunction as a function of time and susceptibility of individual cell types to lifetime exposure to antiretroviral drugs

The outcome of several studies demonstrates that early time-point exposure experiments are not sufficient in studies on ART-induced mitochondrial dysfunction (Fig. 4). For example, neurons might not show mitochondrial alternations after early initiation of treatment but can be affected later in life. In fact, low NAA, previously discussed as a marker of alternations in mitochondrial morphology and HAND onset, was detected in 9 year old HIV positive children but not in 7 year old HIV positive children (Robertson et al. 2018); suggesting that in spite of the initial HIV suppression, neuronal damage might develop at a later time-point. This statement is further supported by the detection of ART-induced mitochondrial toxicity in liver and muscle cells much earlier than in neurons (Zhang et al. 2014) as well as the detection of mtDNA mutations in the cerebral cortex of autopsied HIV-infected patients on ART (Zhang et al. 2012), challenging the findings of previous studies that reported no effects on brain mtDNA for patients that were treated with ART for shorter period of times (Davison et al. 1996). Additionally, other types of cells interacting with the BBB could be exacerbating the effects, as a cross-sectional study on HIV patients taking combination ART therapy found increased break frequency of mtDNA 8-hydroxy-2-deoxyguanosine (8-oxo-dG) in peripheral blood mononuclear cells correlating with chronic systemic structural brain changes and cognitive difficulties (Kallianpur et al. 2016). Hence, when testing for mitochondrial toxicity of novel drugs it is crucial to obtain data on their long-term effects and check the alternations of parameters on the individual cell type level, rather than in whole brain tissue. Moreover, an aspect that should be taken into consideration when detecting cell types vulnerable to ART is their ability to convert the parent drugs into active drug forms. For NRTIs for instance, it is well known that the cells need to express the cell-cycle dependent thymidine kinase 1 and 2 (Bazzoli et al. 2010).

## Toxicity, Autophagy, and Apoptosis

When discussing ART-induced toxicity, it should not be excluded that the HIV infection and HIV proteins (Villeneuve et al. 2016) might be exacerbating the overall toxic effects on mitochondria. Experiments investigating ART and viral effects separately have found that at least part of mitochondrial toxicity stems from the ART independently (Ng et al. 2010). Nevertheless, evidence shows that the common assays, such as MTT, used to confirm toxicity are not sufficient to extrapolate mitochondrial functionality since mitochondria could be affected even if the overall drug toxicity is low. Testing antiretrovirals of the different classes according to their toxicity indexes resulted in an observation that the drug inducing the highest compromise of mitochondrial membrane potential was previously established as the least toxic, whereas the previously established most toxic drugs had variable effects of either decreasing or increasing mitochondrial membrane potential (Robertson et al. 2012). Thereafter, when testing newer antiretrovirals it is essential to conduct stringent mitochondria-specific assays in order to be confident that mitochondrial function is not affected. In addition, it is crucial to test for toxicity when antiretrovirals are used in combinations, i.e., in the form that they are commonly prescribed in the clinic. Moreover, it would be important to understand the mechanisms that explain higher toxicities in certain tissues such as the brain. For instance, the findings of a recent study on prophylactic AZT administration suggests that the mechanism behind increased mitochondrial brain toxicity in some individuals, but not in others, could be explained through differential activity of the ATP-binding cassette efflux transporters which influence the transfer of AZT to the brain. These interactions may be influenced via genetic, pathological or iatrogenic factors, consequently increasing the antiretroviral-induced mitochondrial toxic effects on the brain tissue (Filia et al. 2017).

Interestingly, ART-induced mitochondrial toxicity can be reversible, and it is not as severe as mitochondrial toxicity induced by the HIV proteins that frequently progress to apoptosis, which is then irreversible (Fiala et al. 2004). Recent findings suggest that the lack of activation of apoptosis might be due to a lower level of toxicity of ART which progresses into autophagy but not apoptosis (Gao et al. 2017). Specifically, low doses of nucleoside analogs were found to induce autophagy, while high doses resulted in neuronal apoptosis. The same study reported autophagy activation in autopsy samples from HIV patients and found that the damage-modulated autophagy regulator functioned in a p53 independent manner. However, more research is needed to fully discriminate between the induction of these pathways since antiretrovirals such as Zalcitabine were found to decrease anti-apoptotic proteins and increase pro-apoptotic proteins in addition to the release of cytochrome c, suggesting promotion of apoptosis (Opii et al. 2007).

Our group extensively examined the induction of the ER stress responses and autophagy in response to ART in brain endothelial cells and brain capillaries. Among studied drugs (Efavirenz, Tenofovir, Emtricitabine, Lamivudine, and Indinavir), only Efavirenz increased ER stress via upregulation and activation of the inositol requiring kinase 1 α (IRE1α) and protein kinase-like ER kinase (PERK) signaling. At the same time, Efavirenz diminished autophagic activity, a surprising result as the induction of ER stress is typically linked to enhanced autophagy. These results were confirmed in microvessels of HIV transgenic mice chronically administered with Efavirenz. In a series of further experiments, we identified that Efavirenz dysregulated ER stress and autophagy by blocking the activity of the Beclin-1/Atg14/PI3KIII complex in regard to synthesis of phosphatidylinositol 3-phosphate (PI3P), a process which is linked to the formation of autophagosomes. Because autophagy is a protective mechanism involved in the removal of dysfunctional proteins and/or organelles, its inhibition may contribute to the toxicity of Efavirenz and/or the development of neurodegenerative diseases in HIV patients treated with this drug (Bertrand and Toborek 2015).

## Conditions Exacerbating ART-Induced Mitochondrial Dysfunction

ART exposure induces several factors that can exacerbate their toxicity and contribute to the severity of impaired mitochondrial function. Among these conditions are reactive oxygen species (ROS), nitric oxide (NO), altered expression of growth factors, and calcium signaling. There is extensive evidence for oxidative stress induction in correlation with ART in different brain cell types and as the result of exposure to different antiretroviral drug classes (Mollace et al. 2001; Steiner et al. 2006). The human brain microvascular endothelial cell line (hCMEC/D3) was shown to exhibit increased ROS as well as mitochondrial superoxide levels after exposure to Zidovudine and Indinavir (Prasad et al. 2016), and both of these parameters were also increased due to Tenofovir, Emtricitabine, Ritonavir, and Darunavir (NRTIs, protease, and integrase inhibitors combination) exposure to NPCs (Velichkovska et al. 2018). Furthermore, as a representative of a potent NRTIs, Zalcitabine was found to increase oxidative stress in isolated mitochondria and synaptosomes as indicated by alternations of protein carbonyls and 3-nitrotyrosine levels (Opii et al. 2007). Importantly, these effects were attenuated upon administration of the antioxidant tricyclodecan-9-yl-xanthogenate which can access the brain and also acts as a glutathione mimetic. The endothelial cell-based in-vitro model of the BBB was also found to experience an increase in ROS after exposure to ART combination, and the resulting cytotoxicity was attributed to mitochondrial and oxidative stress mechanisms since mitochondrial potential and ATP levels were altered as well as the levels of glutathione and malondialdehyde (Manda et al. 2011). Pretreatment with a thiol antioxidant N-acetylcysteine amide resulted in attenuation of some of these pro-oxidative effects. Our group recently showed that oxidative stress is a component of the ART-induced mitochondrial dysfunction of NPCs. Importantly, oxidative stress and mitochondrial dysfunction could be prevented by mitochondria-targeted delivery of the antioxidant Coenzyme Q10 (Velichkovska et al. 2018).

Nitric oxide expression was also found to increase in response to ART exposure of glia, the effect that subsequently induced alternations of activity of Complex III and Complex IV (Apostolova et al. 2015b), raising the possibility of nitric oxide to be involved in the mechanisms inhibiting complexes of the electron transport chain. This notion was supported by the observation that inhibition of nitric oxide synthase attenuated ART-induced impairment of mitochondrial function.

Neurons exposed to Didanosine were found to exhibit a decrease in transcript and protein levels of the neurotrophic factor BDNF as well as the BDNF receptor TrkB in Schwann cells (Zhu et al. 2007). More recently, mouse hippocampal tissue exposed to Nevirapine was also found to have decreased expression of BDNF (Zulu et al. 2018). BDNF is a neurotrophic factor which regulates the growth and development of neurons, both of which are impaired under ART. Interestingly, studies have shown that the BDNF receptor has been found to localize on the mitochondrial membrane (Wiedemann et al. 2006), and BDNF was linked to activation of mitochondrial function (El Idrissi and Trenkner 1999) as well as increased respiratory mitochondrial coupling (Markham et al. 2004), emphasizing direct influence on mitochondrial function. Indeed, supplementation with BDNF attenuated the neurotoxic and mitochondrial changes (Zhu et al. 2007).

Calcium signaling also seems to be an important mediator of ART-induced mitochondrial dysfunction. Alterations of calcium signaling may explain the impact of ART on mitochondrial membrane potential and increased secretion of glutamate, which has repeatedly been observed in HAND-related studies. Induced calcium signaling and the consequent opening of the mitochondrial permeability transition pore (mPTP) were found in human astrocytes under exposure of HAND-relevant conditions, such as ART, HIV-1 virions exposure, and inflammation (Nooka and Ghorpade 2017). mPTP opening is detrimental to mitochondria, resulting in the disintegration of their membrane structure, increased ROS concentrations, and depolarization of mitochondrial membrane potential (Görlach et al. 2015). All of these events induce apoptosis (Tsujimoto and Shimizu 2006). ART-induced increase in intracellular calcium levels was also reported to increase glutamate secretion (Malarkey and Parpura 2008), another important factor in the pathogenesis of HAND (Vazquez-Santiago et al. 2014). Importantly, chelation of calcium reversed both mPTP opening (Nooka and Ghorpade 2017) and glutamate release (Bezzi et al. 1998). Hence, mitochondrial dysfunction appears to be closely related to ER stress, suggesting that controlling the ER may minimize, if not eliminate, mitochondrial dysfunction.

## ART Toxicity beyond the BBB

While the bulk of the presented review is concentrated on the cells of the neurovascular unit, ART toxicity is a systemic problem that needs careful consideration. While therapy adherence is primordial to prevent viral rebound, around 30% of patients are forced to change ART regimen to diminish the developing side effects (Troya and Bascunana 2016). Individual differences in susceptibility to antiretroviral toxicity still remain to be elucidated; however, several studies indicate that gene polymorphism may be involved, along with gender and ethnic background (Martin et al. 2004; Haas and Tarr 2015; Dold et al. 2017). The majority of the identified pathways of toxicity presented in this review also applies to cells of other organs. Endothelial dysfunction, dysregulation of ER stress and autophagy pathways, mitochondrial toxicity, calcium imbalance, and several other side effects have also been observed in kidneys, the gut, major arteries, liver, and other organs (Apostolova et al. 2011; Hall et al. 2011; Jones and Nunez 2012; Neuman et al. 2012; Post 2014; Thein et al. 2014; Weiss et al. 2016). These mechanisms of toxicity can lead to gastrointestinal toxicity (nausea, vomiting, and diarrhea), liver toxicity (hyperbilirubinemia and elevated liver enzymes), renal toxicity (elevated creatinine, renal impairment, and tubular dysfunction), cardiovascular toxicity (dyslipidemia, atherosclerosis, and vasculopathy), and other disorders (Reviewed in Troya and Bascunana 2016).

## Conclusions

Balancing the health risks and gains of ART has to be considered in order to maximize the positive effects of therapy. While the most toxic antiretroviral drugs (e.g., Efavirenz) are slowly being taken out of the market, more research is still needed to fully discriminate ART toxicity and its contribution to comorbidities observed in HIV-positive patients, especially in the context of aging, which alters both drug kinetics and susceptibility of the host to ART-induced side effects. The most prominent mechanism of ART toxicity appears to be related to mitochondrial toxicity; therefore, it may be useful to introduce a supplemental therapy that protects against this effect by mitochondria-targeted delivery of antioxidants as recently proposed by our group (Velichkovska et al. 2018).

## References

[CR1] Aging USSSCo (2013) The changing face of HIV/AIDS in America. In: Hearing: older Americans. Washington, D.C.: One Hundred Thirteenth Congress

[CR2] Ahmed D, Roy D, Cassol E (2018). Examining relationships between metabolism and persistent inflammation in HIV patients on Antiretroviral Therapy. Mediat Inflamm.

[CR3] Ajdary M, Moosavi MA, Rahmati M, Falahati M, Mahboubi M, Mandegary A, Jangjoo S, Mohammadinejad R, Varma RS (2018) Health concerns of various nanoparticles: a review of their in vitro and in vivo toxicity. Nanomaterials (Basel) 8 10.3390/nano8090634 PMC6164883 30134524

[CR4] Akay C (2014). Antiretroviral drugs induce oxidative stress and neuronal damage in the central nervous system. J Neurovirol.

[CR5] Alakkas A, Ellis RJ, Watson CW-M, Umlauf A, Heaton RK, Letendre S, Collier A, Marra C, Clifford DB, Gelman B, Sacktor N, Morgello S, Simpson D, McCutchan JA, Kallianpur A, Gianella S, Marcotte T, Grant I, Fennema-Notestine C (2018) White matter damage, neuroinflammation, and neuronal integrity in HAND. J Neurovirol 10.1007/s13365-018-0682-9 PMC6416232 30291567

[CR6] Antiretroviral Therapy Cohort C (2008). Life expectancy of individuals on combination antiretroviral therapy in high-income countries: a collaborative analysis of 14 cohort studies. Lancet.

[CR7] Apostolova N, Gomez-Sucerquia LJ, Gortat A, Blas-Garcia A, Esplugues JV (2011). Compromising mitochondrial function with the antiretroviral drug efavirenz induces cell survival-promoting autophagy. Hepatology.

[CR8] Apostolova N, Funes HA, Blas-Garcia A, Galindo MJ, Alvarez A, Esplugues JV (2015). Efavirenz and the CNS: what we already know and questions that need to be answered. J Antimicrob Chemother.

[CR9] Apostolova N, Funes HA, Blas-Garcia A, Alegre F, Polo M, Esplugues JV (2015). Involvement of nitric oxide in the mitochondrial action of efavirenz: a differential effect on neurons and glial cells. J Infect Dis.

[CR10] Auclair M, Afonso P, Capel E, Caron-Debarle M, Capeau J (2014). Impact of darunavir, atazanavir and lopinavir boosted with ritonavir on cultured human endothelial cells: beneficial effect of pravastatin. Antivir Ther.

[CR11] Barillari G, Iovane A, Bacigalupo I, Labbaye C, Chiozzini C, Sernicola L, Quaranta MT, Falchi M, Sgadari C, Ensoli B (2014). The HIV protease inhibitor indinavir down-regulates the expression of the pro-angiogenic MT1-MMP by human endothelial cells. Angiogenesis.

[CR12] Bates TE, Strangward M, Keelan J, Davey GP, Munro PMG, Clark JB (1996). Inhibition of N-acetylaspartate production. NeuroReport.

[CR13] Baxi SM, Greenblatt RM, Bacchetti P, Scherzer R, Minkoff H, Huang Y, Anastos K, Cohen M, Gange SJ, Young M, Shlipak MG, Gandhi M (2014). Common clinical conditions - age, low BMI, ritonavir use, mild renal impairment - affect tenofovir pharmacokinetics in a large cohort of HIV-infected women. AIDS.

[CR14] Bazzoli C, Jullien V, Le Tiec C, Rey E, Mentré F, Taburet A-M (2010). Intracellular pharmacokinetics of Antiretroviral drugs in HIV-infected patients, and their correlation with drug action. Clin Pharmacokinet.

[CR15] Becker JT, Lopez OL, Dew MA, Aizenstein HJ (2004). Prevalence of cognitive disorders differs as a function of age in HIV virus infection. AIDS.

[CR16] Belgamwar A, Khan S, Yeole P (2018). Intranasal chitosan-g-HPbetaCD nanoparticles of efavirenz for the CNS targeting. Artif Cells Nanomed Biotechnol.

[CR17] Benki-Nugent S, Eshelman C, Wamalwa D, Langat A, Tapia K, Okinyi HM, John-Stewart G (2015). Correlates of age at attainment of developmental milestones in HIV-infected infants receiving early Antiretroviral Therapy. Pediatr Infect Dis J.

[CR18] Bertrand L, Toborek M (2015). Dysregulation of endoplasmic reticulum stress and Autophagic responses by the Antiretroviral drug Efavirenz. Mol Pharmacol.

[CR19] Bertrand L, Nair M, Toborek M (2016). Solving the blood-brain barrier challenge for the effective treatment of HIV replication in the central nervous system. Curr Pharm Des.

[CR20] Bertrand L, Dygert L, Toborek M (2016). Antiretroviral treatment with Efavirenz disrupts the blood-brain barrier integrity and increases stroke severity. Sci Rep.

[CR21] Bertrand L, Cho HJ, Toborek M (2019). Blood-brain barrier pericytes as a target for HIV-1 infection. Brain.

[CR22] Bertrand L, Méroth F, Tournebize M, A. L, Sun E, Toborek M (2019b) Targeting the HIV-infected brain to improve stroke outcome. Nat Commun 10:2009. 10.1038/s41467-019-10046-x PMC6494822 31043599

[CR23] Bezzi P, Carmignoto G, Pasti L, Vesce S, Rossi D, Rizzini BL, Pozzan T, Volterra A (1998). Prostaglandins stimulate calcium-dependent glutamate release in astrocytes. Nature.

[CR24] Bjartmar C, Battistuta J, Terada N, Dupree E, Trapp BD (2002). N-acetylaspartate is an axon-specific marker of mature white matter in vivo: a biochemical and immunohistochemical study on the rat optic nerve. Ann Neurol.

[CR25] Blas-García A, Apostolova N, Ballesteros D, Monleón D, Morales JM, Rocha M, Victor VM, Esplugues JV (2010). Inhibition of mitochondrial function by efavirenz increases lipid content in hepatic cells. Hepatology.

[CR26] Brabers NA, Nottet HS (2006). Role of the pro-inflammatory cytokines TNF-alpha and IL-1beta in HIV-associated dementia. Eur J Clin Investig.

[CR27] Brandmann M, Nehls U, Dringen R (2013). 8-Hydroxy-efavirenz, the primary metabolite of the Antiretroviral drug Efavirenz, stimulates the glycolytic flux in cultured rat astrocytes. Neurochem Res.

[CR28] Cassol E, Misra V, Dutta A, Morgello S, Gabuzda D (2014). Cerebrospinal fluid metabolomics reveals altered waste clearance and accelerated aging in HIV patients with neurocognitive impairment. AIDS.

[CR29] Castro V, Skowronska M, Lombardi J, He J, Seth N, Velichkovska M, Toborek M (2017). Occludin regulates glucose uptake and ATP production in pericytes by influencing AMP-activated protein kinase activity. J Cereb Blood Flow Metab.

[CR30] Chan GN, Patel R, Cummins CL, Bendayan R (2013). Induction of P-glycoprotein by antiretroviral drugs in human brain microvessel endothelial cells. Antimicrob Agents Chemother.

[CR31] Chetty R (2001). Vasculitides associated with HIV infection. J Clin Pathol.

[CR32] Cho HJ, Kuo AM, Bertrand L, Toborek M (2017). HIV alters gap junction-mediated intercellular communication in human brain Pericytes. Front Mol Neurosci.

[CR33] Chow YW, Leong CL, Chow HL, Hooi LS (2007). Lactic acidosis in HIV patients receiving highly active antiretroviral therapy. Med J Malaysia.

[CR34] Chow FC, Regan S, Feske S, Meigs JB, Grinspoon SK, Triant VA (2012). Comparison of ischemic stroke incidence in HIV-infected and non-HIV-infected patients in a US health care system. J Acquir Immune Defic Syndr.

[CR35] Chwiki S, Campos MM, McLaughlin ME, Kleiner DE, Kovacs JA, Morse CG, Abu-Asab MS (2017). Adverse effects of antiretroviral therapy on liver hepatocytes and endothelium in HIV patients: an ultrastructural perspective. Ultrastruct Pathol.

[CR36] Ciavatta VT, Bichler EK, Speigel IA, Elder CC, Teng SL, Tyor WR, García PS (2017). In vitro and ex vivo neurotoxic effects of Efavirenz are greater than those of other common Antiretrovirals. Neurochem Res.

[CR37] Clark AJ, Davis ME (2015). Increased brain uptake of targeted nanoparticles by adding an acid-cleavable linkage between transferrin and the nanoparticle core. Proc Natl Acad Sci U S A.

[CR38] Clifford DB, Ances BM (2013). HIV-associated neurocognitive disorder. Lancet Infect Dis.

[CR39] Cohen J, D’Agostino L, Wilson J, Tuzer F, Torres C (2017) Astrocyte senescence and metabolic changes in response to HIV Antiretroviral Therapy drugs. Front Aging Neurosci 9 10.3389/fnagi.2017.00281 PMC5581874 28900395

[CR40] Cohen J, D'Agostino L, Tuzer F, Torres C (2018). HIV antiretroviral therapy drugs induce premature senescence and altered physiology in HUVECs. Mech Ageing Dev.

[CR41] Cooperman NA, Arnsten JH, Klein RS (2007). Current sexual activity and risky sexual behavior in older men with or at risk for HIV infection. AIDS Educ Prev.

[CR42] Cornford EM, Hyman S (2005). Localization of brain endothelial luminal and abluminal transporters with immunogold electron microscopy. NeuroRx.

[CR43] Crawford KW, Spritzler J, Kalayjian RC, Parsons T, Landay A, Pollard R, Stocker V, Lederman MM, Flexner C, Team ACTP (2010). Age-related changes in plasma concentrations of the HIV protease inhibitor lopinavir. AIDS Res Hum Retrovir.

[CR44] Cysique LA, Brew BJ (2014). The effects of HIV and aging on brain functions: proposing a research framework and update on last 3 years' findings. Curr Opin HIV AIDS.

[CR45] Davison FD, Sweeney BJ, Scaravilli F (1996). Mitochondrial DNA levels in the brain of HIV-positive patients after zidovudine therapy. J Neurol.

[CR46] Deeks SG, Lewin SR, Havlir DV (2013). The end of AIDS: HIV infection as a chronic disease. Lancet.

[CR47] Demougeot C, Garnier P, Mossiat C, Bertrand N, Giroud M, Beley A, Marie C (2001). N-Acetylaspartate, a marker of both cellular dysfunction and neuronal loss: its relevance to studies of acute brain injury. J Neurochem.

[CR48] Dhawan S, Puri RK, Kumar A, Duplan H, Masson JM, Aggarwal BB (1997). Human immunodeficiency virus-1-tat protein induces the cell surface expression of endothelial leukocyte adhesion molecule-1, vascular cell adhesion molecule-1, and intercellular adhesion molecule-1 in human endothelial cells. Blood.

[CR49] Divi RL, Einem TL, Leonard Fletcher SL, Shockley ME, Kuo MM, St Claire MC, Cook A, Nagashima K, Harbaugh SW, Harbaugh JW, Poirier MC (2010). Progressive mitochondrial compromise in brains and livers of Primates exposed in utero to nucleoside reverse transcriptase inhibitors (NRTIs). Toxicol Sci.

[CR50] Dold L, Luda C, Schwarze-Zander C, Boesecke C, Hansel C, Nischalke HD, Lutz P, Mohr R, Wasmuth JC, Strassburg CP, Trebicka J, Rockstroh JK, Spengler U (2017). Genetic polymorphisms associated with fatty liver disease and fibrosis in HIV positive patients receiving combined antiretroviral therapy (cART). PLoS One.

[CR51] Drewe J, Gutmann H, Fricker G, Torok M, Beglinger C, Huwyler J (1999). HIV protease inhibitor ritonavir: a more potent inhibitor of P-glycoprotein than the cyclosporine analog SDZ PSC 833. Biochem Pharmacol.

[CR52] Echeverria P, Gomez-Mora E, Roura S, Bonjoch A, Puig J, Perez-Alvarez N, Bayes-Genis A, Clotet B, Blanco J, Negredo E (2017). Variable endothelial cell function restoration after initiation of two antiretroviral regimens in HIV-infected individuals. J Antimicrob Chemother.

[CR53] El Idrissi A, Trenkner E (1999). Growth factors and taurine protect against excitotoxicity by stabilizing calcium homeostasis and energy metabolism. J Neurosci.

[CR54] Ewings EL, Gerschenson M, St. Claire MC, Nagashima K, Skopets B, Harbaugh SW, Harbaugh JW, Poirier MC (2000). Genotoxic and functional consequences of Transplacental zidovudine exposure in fetal monkey brain mitochondria. J Acquir Immune Defic Syndr.

[CR55] Faltz M, Bergin H, Pilavachi E, Grimwade G, Mabley JG (2017). Effect of the anti-retroviral drugs Efavirenz, Tenofovir and Emtricitabine on endothelial cell function: role of PARP. Cardiovasc Toxicol.

[CR56] Farhadian S, Patel P, Spudich S (2017). Neurological complications of HIV infection. Curr Infect Dis Rep.

[CR57] Ferretti F, Gisslen M, Cinque P, Price RW (2015). Cerebrospinal fluid HIV escape from Antiretroviral Therapy. Curr HIV/AIDS Rep.

[CR58] Ferrucci A, Nonnemacher MR, Wigdahl B (2013). Extracellular HIV-1 viral protein R affects astrocytic glyceraldehyde 3-phosphate dehydrogenase activity and neuronal survival. J Neuro-Oncol.

[CR59] Fiala M, Murphy T, MacDougall J, Yang W, Luque A, Iruela-Arispe L, Cashman J, Buga G, Byrns RE, Barbaro G, Arthos J (2004). HAART drugs induce mitochondrial damage and intercellular gaps and gp120 causes apoptosis. Cardiovasc Toxicol.

[CR60] Filia MF, Marchini T, Minoia JM, Roma MI, De Fino FT, Rubio MC, Copello GJ, Evelson PA, Peroni RN (2017). Induction of ABCG2/BCRP restricts the distribution of zidovudine to the fetal brain in rats. Toxicol Appl Pharmacol.

[CR61] Funes HA, Apostolova N, Alegre F, Blas-Garcia A, Alvarez A, Marti-Cabrera M, Esplugues JV (2014). Neuronal bioenergetics and acute mitochondrial dysfunction: a clue to understanding the central nervous system side effects of Efavirenz. J Infect Dis.

[CR62] Galescu O, Bhangoo A, Ten S (2013). Insulin resistance, lipodystrophy and cardiometabolic syndrome in HIV/AIDS. Rev Endocr Metab Disord.

[CR63] Gao Z, Shan J, Wang B, Qiao L, Chen D, Zhang Y (2017). DRAM is involved in regulating nucleoside analog-induced neuronal autophagy in a p53-independent manner. Mol Neurobiol.

[CR64] Gerschenson M, Nguyen V, Ewings EL, Ceresa A, Shaw JA, St. Claire MC, Nagashima K, Harbaugh SW, Harbaugh JW, Olivero OA, Divi RL, Albert PS, Poirier MC (2004). Mitochondrial toxicity in fetal Erythrocebus patas monkeys exposed Transplacentally to zidovudine plus lamivudine. AIDS Res Hum Retrovir.

[CR65] Görlach A, Bertram K, Hudecova S, Krizanova O (2015). Calcium and ROS: a mutual interplay. Redox Biol.

[CR66] Gross AM, Jaeger PA, Kreisberg JF, Licon K, Jepsen KL, Khosroheidari M, Morsey BM, Swindells S, Shen H, Ng CT, Flagg K, Chen D, Zhang K, Fox HS, Ideker T (2016). Methylome-wide analysis of chronic HIV infection reveals five-year increase in biological age and epigenetic targeting of HLA. Mol Cell.

[CR67] Gu J, Al-Bayati K, Ho EA (2017). Development of antibody-modified chitosan nanoparticles for the targeted delivery of siRNA across the blood-brain barrier as a strategy for inhibiting HIV replication in astrocytes. Drug Deliv Transl Res.

[CR68] Haas DW, Tarr PE (2015). Perspectives on pharmacogenomics of antiretroviral medications and HIV-associated comorbidities. Curr Opin HIV AIDS.

[CR69] Hall AM, Hendry BM, Nitsch D, Connolly JO (2011). Tenofovir-associated kidney toxicity in HIV-infected patients: a review of the evidence. Am J Kidney Dis.

[CR70] Haser GC, Sumpio B (2017). Systemic and cell-specific mechanisms of vasculopathy induced by human immunodeficiency virus and highly active antiretroviral therapy. J Vasc Surg.

[CR71] Hawkins RA, O'Kane RL, Simpson IA, Vina JR (2006). Structure of the blood-brain barrier and its role in the transport of amino acids. J Nutr.

[CR72] Hijmans JG, Stockleman K, Reiakvam W, Levy MV, Brewster LM, Bammert TD, Greiner JJ, Connick E, DeSouza CA (2018). Effects of HIV-1 gp120 and tat on endothelial cell sensescence and senescence-associated microRNAs. Physiol Rep.

[CR73] Hsu DC et al (2018) Central nervous system inflammation and infection during early, nonaccelerated simian-human immunodeficiency virus infection in rhesus macaques. J Virol 92 10.1128/JVI.00222-18 PMC5952152 29563297

[CR74] Huang W, Andras IE, Rha GB, Hennig B, Toborek M (2011). PPARalpha and PPARgamma protect against HIV-1-induced MMP-9 overexpression via caveolae-associated ERK and Akt signaling. FASEB J.

[CR75] Hung K-M, Chen P-C, Hsieh H-C, Calkins MJ (2017). Mitochondrial defects arise from nucleoside/nucleotide reverse transcriptase inhibitors in neurons: potential contribution to HIV-associated neurocognitive disorders. Biochim Biophys Acta (BBA) - Mol Basis Dis.

[CR76] Jones M, Nunez M (2012). Liver toxicity of antiretroviral drugs. Semin Liver Dis.

[CR77] Kallianpur KJ, Gerschenson M, Mitchell BI, LiButti DE, Umaki TM, Ndhlovu LC, Nakamoto BK, Chow DC, Shikuma CM (2016). Oxidative mitochondrial DNA damage in peripheral blood mononuclear cells is associated with reduced volumes of hippocampus and subcortical gray matter in chronically HIV-infected patients. Mitochondrion.

[CR78] Kamei N, Yamaoka A, Fukuyama Y, Itokazu R, Takeda-Morishita M (2018). Noncovalent strategy with cell-penetrating peptides to facilitate the brain delivery of insulin through the blood-brain barrier. Biol Pharm Bull.

[CR79] Kamtchum-Tatuene J, Mwandumba H, Al-Bayati Z, Flatley J, Griffiths M, Solomon T, Benjamin L (2019). HIV is associated with endothelial activation despite ART, in a sub-Saharan African setting. Neurol Neuroimmunol Neuroinflamm.

[CR80] Karbowski M, Youle RJ (2003). Dynamics of mitochondrial morphology in healthy cells and during apoptosis. Cell Death Differ.

[CR81] Kebodeaux CD, Wilson AG, Smith DL, Vouri SM (2013). A review of cardiovascular and renal function monitoring: a consideration of older adults with HIV. HIV AIDS (Auckl).

[CR82] Lake JE, Currier JS (2013). Metabolic disease in HIV infection. Lancet Infect Dis.

[CR83] Latronico T, Pati I, Ciavarella R, Fasano A, Mengoni F, Lichtner M, Vullo V, Mastroianni CM, Liuzzi GM (2018). In vitro effect of antiretroviral drugs on cultured primary astrocytes: analysis of neurotoxicity and matrix metalloproteinase inhibition. J Neurochem.

[CR84] Lee YW, Kuhn H, Hennig B, Neish AS, Toborek M (2001). IL-4-induced oxidative stress upregulates VCAM-1 gene expression in human endothelial cells. J Mol Cell Cardiol.

[CR85] Lee YW, Eum SY, Nath A, Toborek M (2004). Estrogen-mediated protection against HIV tat protein-induced inflammatory pathways in human vascular endothelial cells. Cardiovasc Res.

[CR86] Lewis W, Dalakas MC (1995). Mitochondrial toxicity of antiviral drugs. Nat Med.

[CR87] Li S, Wu Y, Keating SM, Du H, Sammet CL, Zadikoff C, Mahadevia R, Epstein LG, Ragin AB (2013). Matrix metalloproteinase levels in early HIV infection and relation to in vivo brain status. J Neuro-Oncol.

[CR88] Lindén M, Nelson BD, Leterrier JF (1989). The specific binding of the microtubule-associated protein 2 (MAP2) to the outer membrane of rat brain mitochondria. Biochem J.

[CR89] Liu Y, Shim Park E, Gibbons AT, Shide ED, Divi RL, Woodward RA, Poirier MC (2016). Mitochondrial compromise in 3-year old patas monkeys exposedin uteroto human-equivalent antiretroviral therapies. Environ Mol Mutagen.

[CR90] Malarkey EB, Parpura V (2008). Mechanisms of glutamate release from astrocytes. Neurochem Int.

[CR91] Manda KR, Banerjee A, Banks WA, Ercal N (2011). Highly active antiretroviral therapy drug combination induces oxidative stress and mitochondrial dysfunction in immortalized human blood–brain barrier endothelial cells. Free Radic Biol Med.

[CR92] Markham A, Cameron I, Franklin P, Spedding M (2004). BDNF increases rat brain mitochondrial respiratory coupling at complex I, but not complex II. Eur J Neurosci.

[CR93] Martin AM, Nolan D, Gaudieri S, Phillips E, Mallal S (2004). Pharmacogenetics of antiretroviral therapy: genetic variation of response and toxicity. Pharmacogenomics.

[CR94] Martinez-Picado J, Deeks SG (2016). Persistent HIV-1 replication during antiretroviral therapy. Curr Opin HIV AIDS.

[CR95] Marzolini C, Back D, Weber R, Furrer H, Cavassini M, Calmy A, Vernazza P, Bernasconi E, Khoo S, Battegay M, Elzi L, Swiss HIVCSM (2011). Ageing with HIV: medication use and risk for potential drug-drug interactions. J Antimicrob Chemother.

[CR96] Marzolini C, Mueller R, Li-Blatter X, Battegay M, Seelig A (2013). The brain entry of HIV-1 protease inhibitors is facilitated when used in combination. Mol Pharm.

[CR97] Mata-Marin JA, Mendez-Cruz R, Arroyo-Anduiza CI, Mata-Marin LA, Gaytan-Martinez J, Asbun-Bojalil J (2013). Effect of antiretroviral therapy on inflammatory markers of endothelial dysfunction in HIV treatment-naive infected patients. J Med Virol.

[CR98] McRae M (2016). HIV and viral protein effects on the blood brain barrier. Tissue Barriers.

[CR99] Modicanapolitano JS (1993). AZT causes tissue-specific inhibition of mitochondrial bioenergetic function. Biochem Biophys Res Commun.

[CR100] Moffett JR, Aryan Namboodiri MA, Cangro CB, Neale JH (1991). Immunohistochemical localization of N-acetylaspartate in rat brain. NeuroReport.

[CR101] Moketla MB, Wadley AL, Kamerman P, de Assis Rosa D (2018). Pharmacogenetic variation influences sensory neuropathy occurrence in southern Africans treated with stavudine-containing antiretroviral therapy. PLoS One.

[CR102] Mollace V, Nottet HSLM, Clayette P, Turco MC, Muscoli C, Salvemini D, Perno CF (2001). Oxidative stress and neuroAIDS: triggers, modulators and novel antioxidants. Trends Neurosci.

[CR103] Mondal D, Pradhan L, Ali M, Agrawal KC (2004). HAART drugs induce oxidative stress in human endothelial cells and increase endothelial recruitment of mononuclear cells: exacerbation by inflammatory cytokines and amelioration by antioxidants. Cardiovasc Toxicol.

[CR104] Morgello S, Murray J, Van Der Elst S, Byrd D (2014). HCV, but not HIV, is a risk factor for cerebral small vessel disease. Neurol Neuroimmunol Neuroinflamm.

[CR105] Nakada K, Inoue K, Ono T, Isobe K, Ogura A, Goto YI, Nonaka I, Hayashi JI (2001). Inter-mitochondrial complementation: mitochondria-specific system preventing mice from expression of disease phenotypes by mutant mtDNA. Nat Med.

[CR106] Neuman MG, Schneider M, Nanau RM, Parry C (2012). HIV-Antiretroviral Therapy induced liver, gastrointestinal, and pancreatic injury. Int J Hepatol.

[CR107] Ng K, Kumar K, Brew B, Burke D (2010). Axonal excitability in viral polyneuropathy and nucleoside neuropathy in HIV patients. J Neurol Neurosurg Psychiatry.

[CR108] Nooka S, Ghorpade A (2017). HIV-1-associated inflammation and antiretroviral therapy regulate astrocyte endoplasmic reticulum stress responses. Cell Death Dis.

[CR109] Oliveira MF, Chaillon A, Nakazawa M, Vargas M, Letendre SL, Strain MC, Ellis RJ, Morris S, Little SJ, Smith DM, Gianella S (2017). Early Antiretroviral Therapy is associated with lower HIV DNA molecular diversity and lower inflammation in cerebrospinal fluid but does not prevent the establishment of compartmentalized HIV DNA populations. PLoS Pathog.

[CR110] Opii WO, Sultana R, Abdul HM, Ansari MA, Nath A, Butterfield DA (2007). Oxidative stress and toxicity induced by the nucleoside reverse transcriptase inhibitor (NRTI)—2′,3′-dideoxycytidine (ddC): relevance to HIV-dementia. Exp Neurol.

[CR111] Palmer TD, Willhoite AR, Gage FH (2000). Vascular niche for adult hippocampal neurogenesis. J Comp Neurol.

[CR112] Park IW, Wang JF, Groopman JE (2001). HIV-1 Tat promotes monocyte chemoattractant protein-1 secretion followed by transmigration of monocytes. Blood.

[CR113] Park M, Kim HJ, Lim B, Wylegala A, Toborek M (2013). Methamphetamine-induced occludin endocytosis is mediated by the Arp2/3 complex-regulated actin rearrangement. J Biol Chem.

[CR114] Pathai S, Bajillan H, Landay AL, High KP (2014). Is HIV a model of accelerated or accentuated aging?. J Gerontol A Biol Sci Med Sci.

[CR115] Peluso MJ, Meyerhoff DJ, Price RW, Peterson J, Lee E, Young AC, Walter R, Fuchs D, Brew BJ, Cinque P, Robertson K, Hagberg L, Zetterberg H, Gisslen M, Spudich S (2013). Cerebrospinal fluid and neuroimaging biomarker abnormalities suggest early neurological injury in a subset of individuals during primary HIV infection. J Infect Dis.

[CR116] Post F (2014). Adverse events: ART and the kidney: alterations in renal function and renal toxicity. J Int AIDS Soc.

[CR117] Prasad S, Sajja RK, Kaisar MA, Cucullo L (2016). Hyperglycemia exacerbates antiretroviral drug combination induced blood-brain barrier endothelial toxicity. Neurotoxicology.

[CR118] Pu H, Tian J, Andras IE, Hayashi K, Flora G, Hennig B, Toborek M (2005). HIV-1 Tat protein-induced alterations of ZO-1 expression are mediated by redox-regulated ERK 1/2 activation. J Cereb Blood Flow Metab.

[CR119] Qosa H, Miller DS, Pasinelli P, Trotti D (2015). Regulation of ABC efflux transporters at blood-brain barrier in health and neurological disorders. Brain Res.

[CR120] Ramirez-Castillejo C, Sanchez-Sanchez F, Andreu-Agullo C, Ferron SR, Aroca-Aguilar JD, Sanchez P, Mira H, Escribano J, Farinas I (2006). Pigment epithelium-derived factor is a niche signal for neural stem cell renewal. Nat Neurosci.

[CR121] Riquelme PA, Drapeau E, Doetsch F (2008). Brain micro-ecologies: neural stem cell niches in the adult mammalian brain. Philos Trans R Soc Lond Ser B Biol Sci.

[CR122] Robertson FC, Holmes MJ, Cotton MF, Dobbels E, Little F, Laughton B, van der Kouwe AJW, Meintjes EM (2018) Perinatal HIV Infection or Exposure Is Associated With Low N-Acetylaspartate and Glutamate in Basal Ganglia at Age 9 but Not 7 Years. Front Hum Neurosci 12:145.Z 10.3389/fnhum.2018.00145 PMC5949349 29867401

[CR123] Robertson K, Liner J, Meeker RB (2012). Antiretroviral neurotoxicity. J Neurovirol.

[CR124] Robillard KR, Chan GN, Zhang G, la Porte C, Cameron W, Bendayan R (2014). Role of P-glycoprotein in the distribution of the HIV protease inhibitor atazanavir in the brain and male genital tract. Antimicrob Agents Chemother.

[CR125] Ross AC, Rizk N, O'Riordan MA, Dogra V, El-Bejjani D, Storer N, Harrill D, Tungsiripat M, Adell J, McComsey GA (2009). Relationship between inflammatory markers, endothelial activation markers, and carotid intima-media thickness in HIV-infected patients receiving antiretroviral therapy. Clin Infect Dis.

[CR126] Roy U, Bulot C, Honer zu Bentrup K, Mondal D (2013). Specific increase in MDR1 mediated drug-efflux in human brain endothelial cells following co-exposure to HIV-1 and saquinavir. PLoS One.

[CR127] Roy U, Drozd V, Durygin A, Rodriguez J, Barber P, Atluri V, Liu X, Voss TG, Saxena S, Nair M (2018). Characterization of Nanodiamond-based anti-HIV drug delivery to the brain. Sci Rep.

[CR128] Sailasuta N, Ananworanich J, Lerdlum S, Sithinamsuwan P, Fletcher JL, Tipsuk S, Pothisri M, Jadwattanakul T, Jirajariyavej S, Chalermchai T, Catella S, Busovaca E, Desai A, Paul R, Valcour V, Group SS (2016). Neuronal-glia markers by magnetic resonance spectroscopy in HIV before and after combination Antiretroviral Therapy. J Acquir Immune Defic Syndr.

[CR129] Samji H (2013). Closing the gap: increases in life expectancy among treated HIV-positive individuals in the United States and Canada. PLoS One.

[CR130] Saribas AS, Khalili K, Sariyer IK (2015). Dysregulation of autophagy by HIV-1 Nef in human astrocytes. Cell Cycle.

[CR131] Schapira AHV (2010). Complex I: inhibitors, inhibition and neurodegeneration. Exp Neurol.

[CR132] Schweinsburg BC, Taylor MJ, Alhassoon OM, Gonzalez R, Brown GG, Ellis RJ, Letendre S, Videen JS, McCutchan JA, Patterson TL, Grant I, the HG (2005). Brain mitochondrial injury in human immunodeficiency virus–seropositive (HIV+) individuals taking nucleoside reverse transcriptase inhibitors. J Neurovirol.

[CR133] Shah A, Gangwani MR, Chaudhari NS, Glazyrin A, Bhat HK, Kumar A (2016). Neurotoxicity in the Post-HAART era: caution for the Antiretroviral therapeutics. Neurotox Res.

[CR134] Shankar SS, Dube MP (2004). Clinical aspects of endothelial dysfunction associated with human immunodeficiency virus infection and antiretroviral agents. Cardiovasc Toxicol.

[CR135] Shen Q, Goderie SK, Jin L, Karanth N, Sun Y, Abramova N, Vincent P, Pumiglia K, Temple S (2004). Endothelial cells stimulate self-renewal and expand neurogenesis of neural stem cells. Science.

[CR136] Shen Q, Wang Y, Kokovay E, Lin G, Chuang SM, Goderie SK, Roysam B, Temple S (2008). Adult SVZ stem cells lie in a vascular niche: a quantitative analysis of niche cell-cell interactions. Cell Stem Cell.

[CR137] Sibiude J, Warszawski J, Blanche S (2015). Tolerance of the newborn to antiretroviral drug exposurein utero. Expert Opin Drug Saf.

[CR138] Sibiude J, Le Chenadec J, Bonnet D, Tubiana R, Faye A, Dollfus C, Mandelbrot L, Delmas S, Lelong N, Khoshnood B, Warszawski J, Blanche S (2015). In utero exposure to zidovudine and heart anomalies in the ANRS French perinatal Cohort and the nested PRIMEVA randomized trial. Clin Infect Dis.

[CR139] Simmons ML, Frondoza CG, Coyle JT (1991). Immunocytochemical localization of N-acetyl-aspartate with monoclonal antibodies. Neuroscience.

[CR140] Soontornniyomkij V, Umlauf A, Chung SA, Cochran ML, Soontornniyomkij B, Gouaux B, Toperoff W, Moore DJ, Masliah E, Ellis RJ, Grant I, Achim CL (2014). HIV protease inhibitor exposure predicts cerebral small vessel disease. AIDS.

[CR141] Soontornniyomkij V, Umlauf A, Soontornniyomkij B, Gouaux B, Ellis RJ, Levine AJ, Moore DJ, Letendre SL (2018) Association of antiretroviral therapy with brain aging changes among HIV-infected adults. AIDS 32:2005–2015 10.1097/QAD.0000000000001927 PMC6115290 29912063

[CR142] Stauch KL, Emanuel K, Lamberty BG, Morsey B, Fox HS (2017). Central nervous system-penetrating antiretrovirals impair energetic reserve in striatal nerve terminals. J Neurovirol.

[CR143] Steiner J, Haughey N, Li W, Venkatesan A, Anderson C, Reid R, Malpica T, Pocernich C, Butterfield DA, Nath A (2006). Oxidative stress and therapeutic approaches in HIV dementia. Antioxid Redox Signal.

[CR144] Stern AL, Lee RN, Panvelker N, Li J, Harowitz J, Jordan-Sciutto KL, Akay-Espinoza C (2018). Differential effects of Antiretroviral drugs on neurons in vitro: roles for oxidative stress and integrated stress response. J NeuroImmune Pharmacol.

[CR145] Strehlau R, Kuhn L, Abrams EJ, Coovadia A (2016). HIV-associated neurodevelopmental delay: prevalence, predictors and persistence in relation to antiretroviral therapy initiation and viral suppression. Child Care Health Dev.

[CR146] Sturdevant CB, Joseph SB, Schnell G, Price RW, Swanstrom R, Spudich S (2015). Compartmentalized replication of R5 T cell-tropic HIV-1 in the central nervous system early in the course of infection. PLoS Pathog.

[CR147] Su B, Wang X, Zheng L, Perry G, Smith MA, Zhu X (2010). Abnormal mitochondrial dynamics and neurodegenerative diseases. Biochim Biophys Acta (BBA) - Mol Basis Dis.

[CR148] Teng H, Zhang ZG, Wang L, Zhang RL, Zhang L, Morris D, Gregg SR, Wu Z, Jiang A, Lu M, Zlokovic BV, Chopp M (2008). Coupling of angiogenesis and neurogenesis in cultured endothelial cells and neural progenitor cells after stroke. J Cereb Blood Flow Metab.

[CR149] Thein P, Kalinec GM, Park C, Kalinec F (2014). In vitro assessment of antiretroviral drugs demonstrates potential for ototoxicity. Hear Res.

[CR150] Toborek M, Barger SW, Mattson MP, McClain CJ, Hennig B (1995). Role of glutathione redox cycle in TNF-alpha-mediated endothelial cell dysfunction. Atherosclerosis.

[CR151] Toborek M, Lee YW, Pu H, Malecki A, Flora G, Garrido R, Hennig B, Bauer HC, Nath A (2003). HIV-tat protein induces oxidative and inflammatory pathways in brain endothelium. J Neurochem.

[CR152] Toborek M, Lee YW, Flora G, Pu H, Andras IE, Wylegala E, Hennig B, Nath A (2005). Mechanisms of the blood-brain barrier disruption in HIV-1 infection. Cell Mol Neurobiol.

[CR153] Tovar-y-Romo LB, Bumpus NN, Pomerantz D, Avery LB, Sacktor N, McArthur JC, Haughey NJ (2012). Dendritic spine injury induced by the 8-Hydroxy metabolite of Efavirenz. J Pharmacol Exp Ther.

[CR154] Troya J, Bascunana J (2016). Safety and tolerability: current challenges to Antiretroviral Therapy for the long-term management of HIV infection. AIDS Rev.

[CR155] Tsujimoto Y, Shimizu S (2006). Role of the mitochondrial membrane permeability transition in cell death. Apoptosis.

[CR156] Vazquez-Santiago FJ, Noel RJ, Porter JT, Rivera-Amill V (2014). Glutamate metabolism and HIV-associated neurocognitive disorders. J Neuro-Oncol.

[CR157] Velichkovska M, Surnar B, Nair M, Dhar S, Toborek M (2018). Targeted mitochondrial COQ10 delivery attenuates Antiretroviral-drug-induced senescence of neural progenitor cells. Mol Pharm.

[CR158] Villeneuve LM, Purnell PR, Stauch KL, Callen SE, Buch SJ, Fox HS (2016). HIV-1 transgenic rats display mitochondrial abnormalities consistent with abnormal energy generation and distribution. J Neurovirol.

[CR159] Warriner AH, Burkholder GA, Overton ET (2014). HIV-related metabolic comorbidities in the current ART era. Infect Dis Clin N Am.

[CR160] Weiss M, Kost B, Renner-Muller I, Wolf E, Mylonas I, Bruning A (2016). Efavirenz causes oxidative stress, endoplasmic reticulum stress, and autophagy in endothelial cells. Cardiovasc Toxicol.

[CR161] Wiedemann F, Siemen D, Mawrin C, Horn T, Dietzmann K (2006). The neurotrophin receptor TrkB is colocalized to mitochondrial membranes. Int J Biochem Cell Biol.

[CR162] Williams JL, Holman DW, Klein RS (2014). Chemokines in the balance: maintenance of homeostasis and protection at CNS barriers. Front Cell Neurosci.

[CR163] Winston A, Underwood J (2015). Emerging concepts on the use of antiretroviral therapy in older adults living with HIV infection. Curr Opin Infect Dis.

[CR164] Winston A, Duncombe C, Li PC, Gill JM, Kerr SJ, Puls R, Petoumenos K, Taylor-Robinson SD, Emery S, Cooper DA, Altair Study G (2010). Does choice of combination antiretroviral therapy (cART) alter changes in cerebral function testing after 48 weeks in treatment-naive, HIV-1-infected individuals commencing cART? A randomized, controlled study. Clin Infect Dis.

[CR165] Wolf K, Tsakiris DA, Weber R, Erb P, Battegay M, Swiss HIVCS (2002). Antiretroviral therapy reduces markers of endothelial and coagulation activation in patients infected with human immunodeficiency virus type 1. J Infect Dis.

[CR166] Wright PW, Vaida FF, Fernandez RJ, Rutlin J, Price RW, Lee E, Peterson J, Fuchs D, Shimony JS, Robertson KR, Walter R, Meyerhoff DJ, Spudich S, Ances BM (2015). Cerebral white matter integrity during primary HIV infection. AIDS.

[CR167] Younas M, Psomas C, Reynes J, Corbeau P (2016). Immune activation in the course of HIV-1 infection: causes, phenotypes and persistence under therapy. HIV Med.

[CR168] Young AC, Yiannoutsos CT, Hegde M, Lee E, Peterson J, Walter R, Price RW, Meyerhoff DJ, Spudich S (2014). Cerebral metabolite changes prior to and after antiretroviral therapy in primary HIV infection. Neurology.

[CR169] Yuan BO, Zhao Y, Dong S, Sun Y, Hao F, Xie J, Teng L, Lee RJ, Fu Y, Bi YE (2019). Cell-penetrating peptide-coated liposomes for drug delivery across the blood-brain barrier. Anticancer Res.

[CR170] Zhang Y, Wang M, Li H, Zhang H, Shi Y, Wei F, Liu D, Liu K, Chen D (2012). Accumulation of nuclear and mitochondrial DNA damage in the frontal cortex cells of patients with HIV-associated neurocognitive disorders. Brain Res.

[CR171] Zhang Y, Song F, Gao Z, Ding W, Qiao L, Yang S, Chen X, Jin R, Chen D (2014). Long-term exposure of mice to nucleoside analogues disrupts mitochondrial DNA maintenance in cortical neurons. PLoS One.

[CR172] Zhang Y, Wang B, Liang Q, Qiao L, Xu B, Zhang H, Yang S, Chen J, Guo H, Wu J, Chen D (2015). Mitochondrial DNA D-loop AG/TC transition mutation in cortical neurons of mice after long-term exposure to nucleoside analogues. J Neurovirol.

[CR173] Zhong Y, Zhang B, Eum SY, Toborek M (2012). HIV-1 tat triggers nuclear localization of ZO-1 via rho signaling and cAMP response element-binding protein activation. J Neurosci.

[CR174] Zhu Y, Antony JM, Martinez JA, Glerum DM, Brussee V, Hoke A, Zochodne D, Power C (2007). Didanosine causes sensory neuropathy in an HIV/AIDS animal model: impaired mitochondrial and neurotrophic factor gene expression. Brain.

[CR175] Zulu SS, Simola N, Mabandla MV, Daniels WMU (2018). Effect of long-term administration of antiretroviral drugs (Tenofovir and Nevirapine) on neuroinflammation and neuroplasticity in mouse hippocampi. J Chem Neuroanat.

